# Supplementary material for: Mitochondrial Phylogenomics Suggests Complex Evolutionary Pattern of Pronotal Foliaceous Mimicry in Hierodulinae (Mantodea: Mantidae), with Description of a New Species of Rhombodera Burmeister, 1838 from China
Source: Insects. 2022 Aug 9;13(8):715. doi: 10.3390/insects13080715 (PMC9409270; doi:10.3390/insects13080715)
Supplement: Supplementary file 1 [file insects-13-00715-s001.zip › insects-1823606-supplementary.pdf]

Supplementary materials

Table S1. Taxa used in present study from GenBank.

	Family	Species	GenBank number
**Ingroup**			
	Mantidae	*Mekongomantis quinquespinosa*	MN267041
		Hierodula membranacea	KR703239
		Hierodula membranacea	NC_048984
		Hierodula patellifera	KX091856
		Hierodula patellifera	KX611803
		Hierodula patellifera	NC_034283
		Rhombomantis fusca	KY689124
		Rhombomantis fusca	NC_034282
		Rhombodera latipronotum	KX091864
		Rhombodera valida	NC_034284
		Hierodula chinensis	KY689112
		Rhombodera zhangi	MW357299
		Rhombodera longa	MT110155
		Pseudovates peruviana	MW357301
		Sphodromantis lineola	NC_037204
		Stagmatoptera biocellata	MW357302
		Statilia sp.	KU201316
		Statilia maculata	KX900484
		Tarachomantis alaotrana	NC_056875
		Mantis religiosa	MN356097
		Tenodera sinensis	KY689132
	Hymenopodidae	*Acromantis hesione*	KX434865
		Anaxarcha sinensis	KX434866
		Ceratomantis saussurii	KX091850
		Creobroter elongata	KX091851
		Creobroter gemmatus	KU201319
		Astyliasula hoffmanni	KX434859
		Hestiasula sp.	KX091855
		Hymenopus coronatus	MZ573776
		Phyllothelys shaanxiense	KX091863
**Outgroup**			
	Toxoderidae	*Toxodera hauseri*	KX434837
		Stenotoxodera porioni	KY689118
	Amelidae	*Yersinia mexicana*	MW357303

Table S2. Collection information and accession number of the ingroups species sequenced in the present study.

Species	Locality	Time	Identification	Accession
Hierodula chinensis	Qingchengshan, Sichuan China	2018-IV-16	[37]	OP108446
Hierodula confusa	Menglun, Mengla, Xishuangbanna, Yunnan, China	2018-VII-1	[36]	OP168273
Hierodula jianfenglingensis	Ledong Li, Hainan, China	2020-XII-19	[37]	OP168274
Hierodula latipennis	Yiwu, Mengla, Xishuangbanna, Yunnan, China	2018-VII-2	[12]	OP168275
Hierodula membranacea	India	2021-XII-12	[10]	OP168276
Rhombodera kirbyi	Indonesia	2020-IV	[38]	OP168284
Rhombodera latipronotum	Menglun, Yunnan, China	2021-XI-21	[39]	OP168278
Rhombodera latipronotum	Fangchenggang, Guangxi, China	2020-IV	[39]	OP168277
Rhombodera longa	Xishuangbanna Dai, Yunnan, China	2021-IV-20	[12]	OP168279
Rhombomantis longipennis	Dehong, Yingjiang, Yunnan China	2021-VII-1	[12]	OP168283
Rhombodera megaera	Muang Krabi, Thailand	2019-I-14	[10]	OP168287
Rhombodera hyalina	Qinzhou, Guangxi, China	2020-XII-17	This study	OP168286
Rhombodera stalii	Java, Indonesia	2019-XII-8	[4]	OP168280
Rhombodera valida	Mengla, Xishuangbanna, Yunnan, China	2017-IV-19	[10]	OP168282
Rhombodera valida	Java, Indonesia	2019-XII	[10]	OP168281
Rhombodera zhangi	Dehong, Yingjiang, Yunnan, China	2021-VIII	[40]	OP168285

Additional Material Examined

Rhombodera valida Burmeister, 1838

Material examined. 1m#, CHINA, Mengla, Xishuangbanna, Yunnan, 2017-iv-19, leg. Chao Wu; 2f#, Jinghong, Xishuangbanna, Yunnan, 2021-x-26, leg. Guozhong Yang; 1m#, Java, Indonesia, 2019-xii.

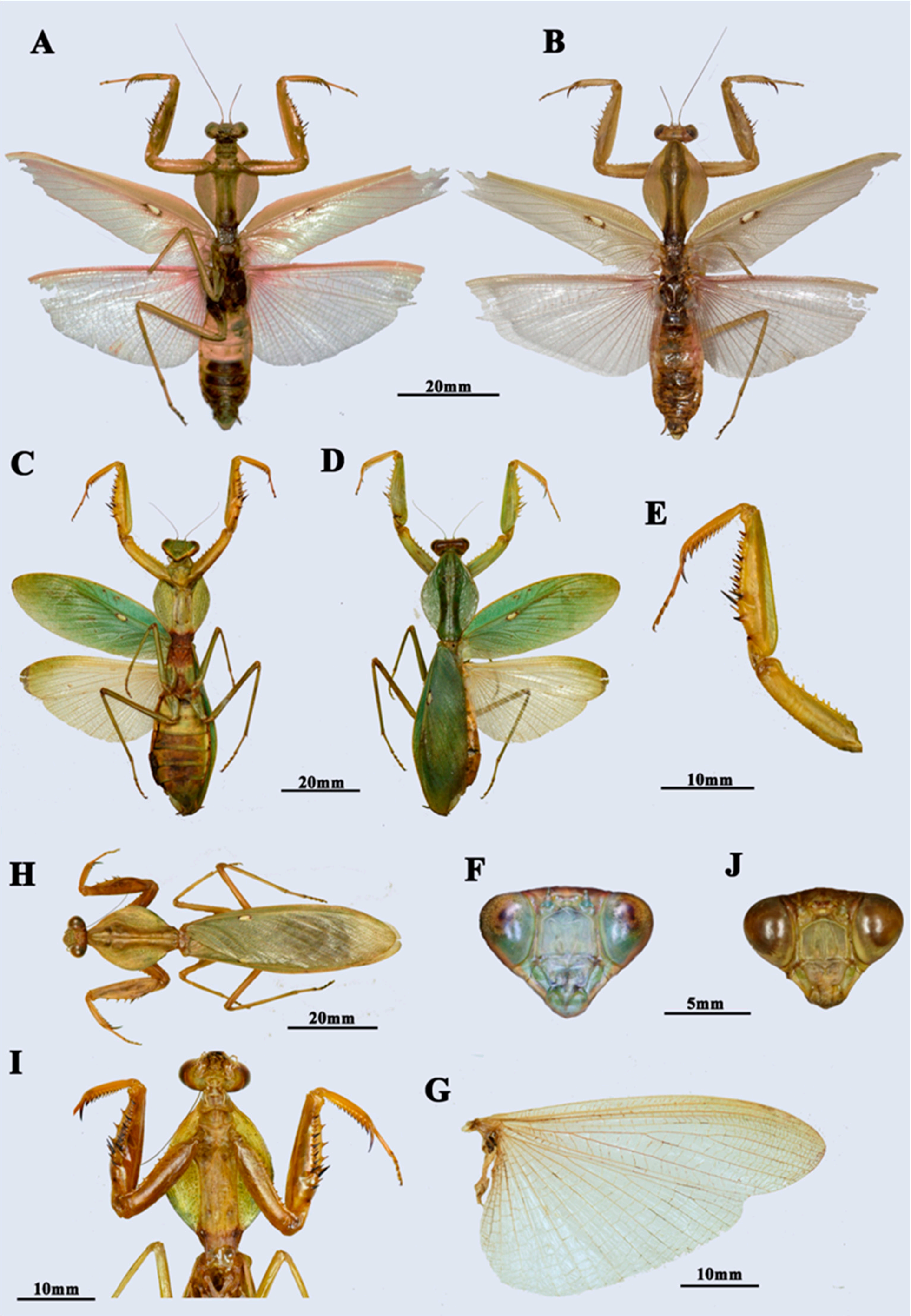

Figure S1. *Rhombodera valida*. A–B. *R. valida* from Java, Indonesia, male. A. Ventral view. B. Dorsal view. C–F. *R. valida* from Yunnan, China, female. C. Ventral view. D. Dorsal view. E. Ventral view of the forelegs. F. Frontal view of head. J–G. *R. valida* from Yunnan, China, male. J. Frontal view of the head. H. Dorsal view. I. Ventral view. G. Hindwing.

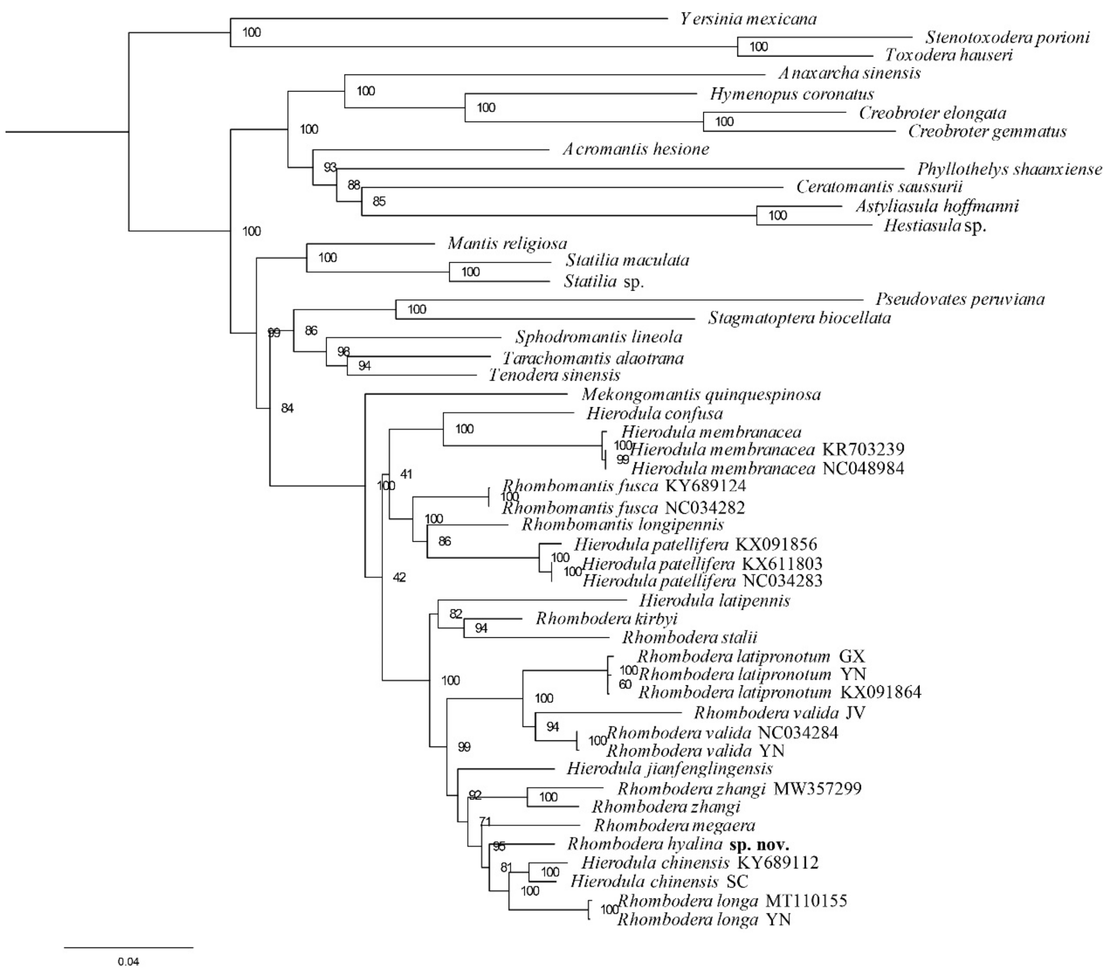

Figure S2. Topology of the ML analysis from AA-no partition dataset.

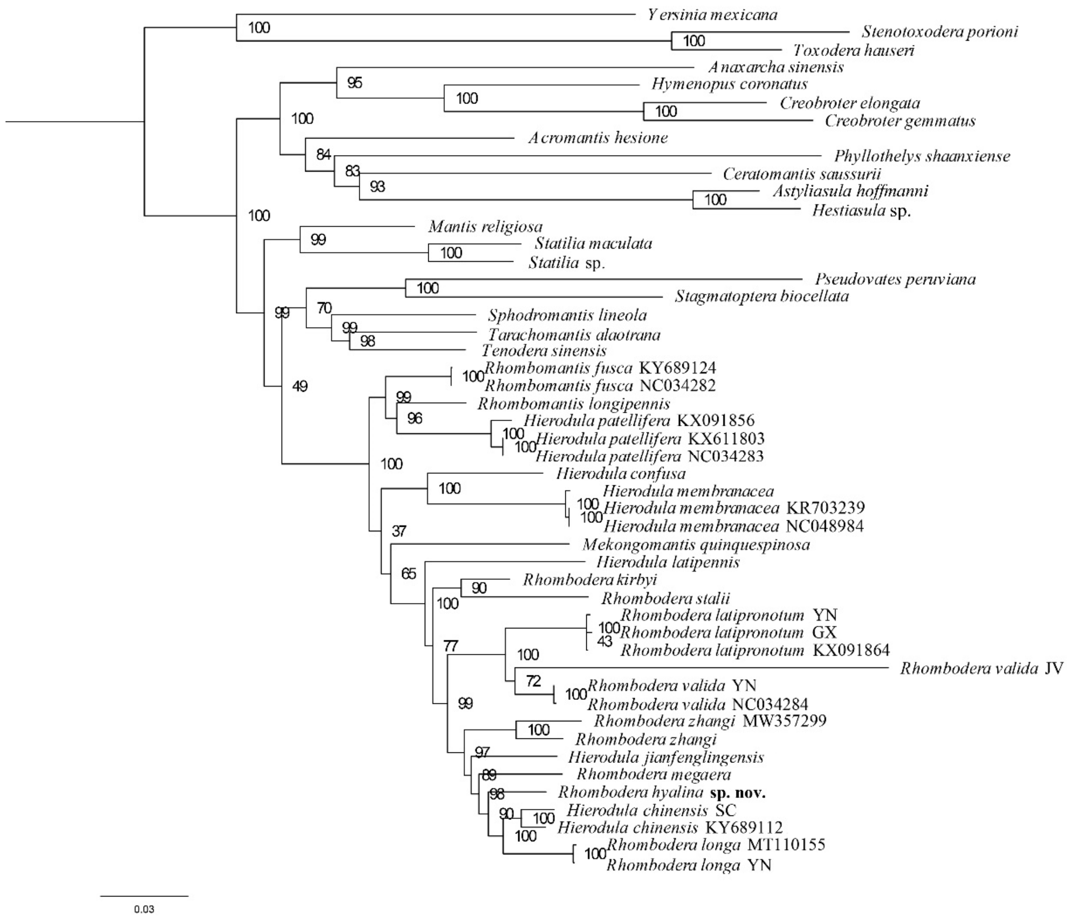

Figure S3. Topology of the ML analysis from AA-gene partition dataset.

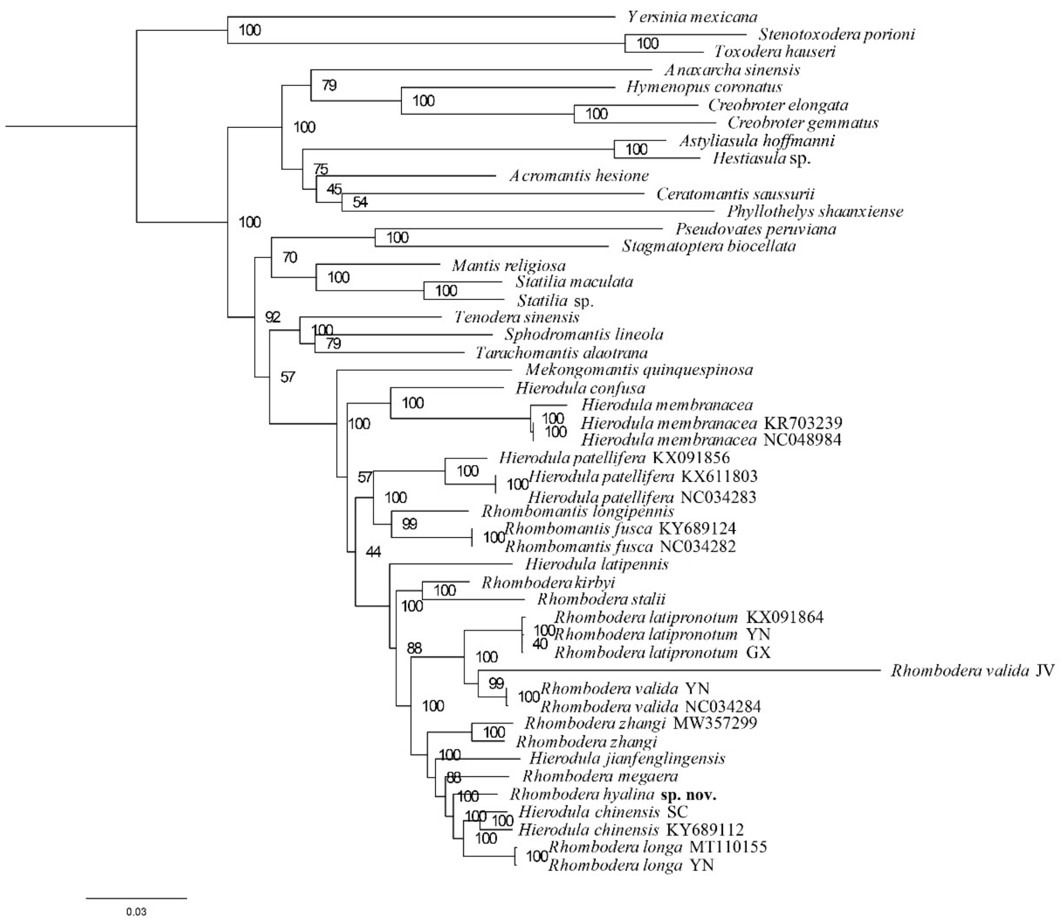

Figure S4. Topology of the ML analysis from PCG12RNA-gene partition dataset.

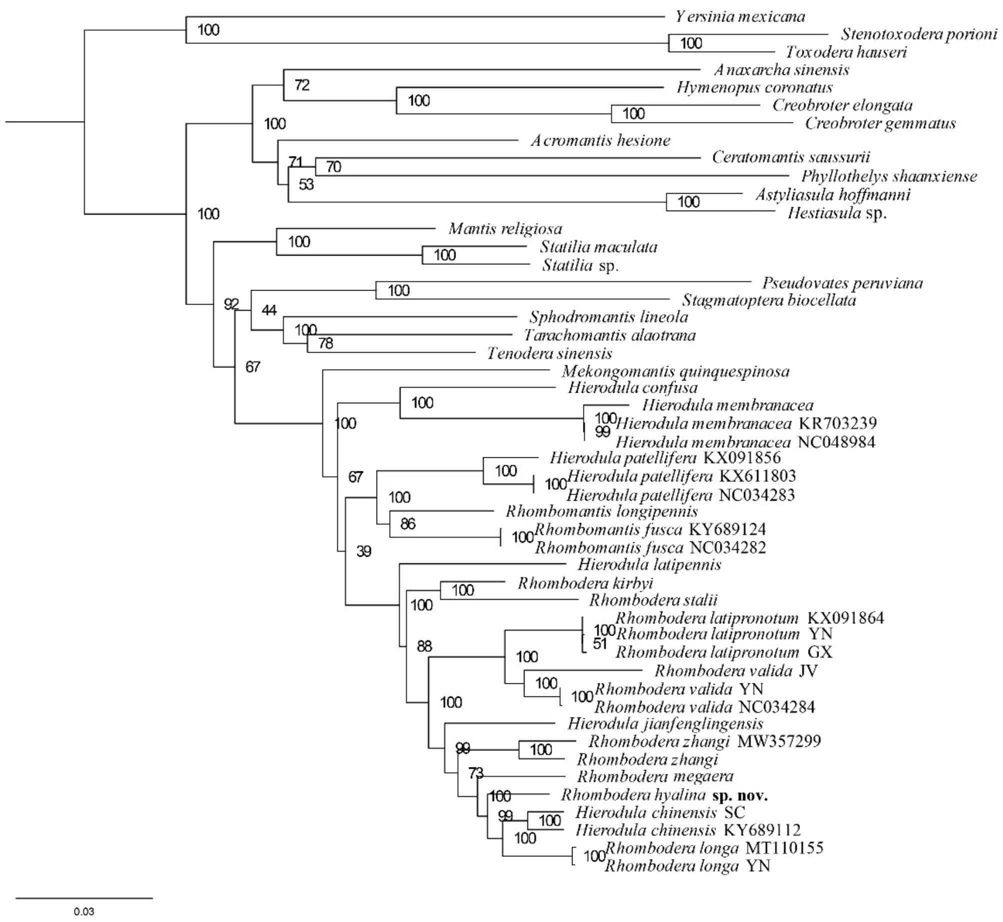

Figure S5. Topology of the ML analysis from PCG12RNA-no partition dataset.

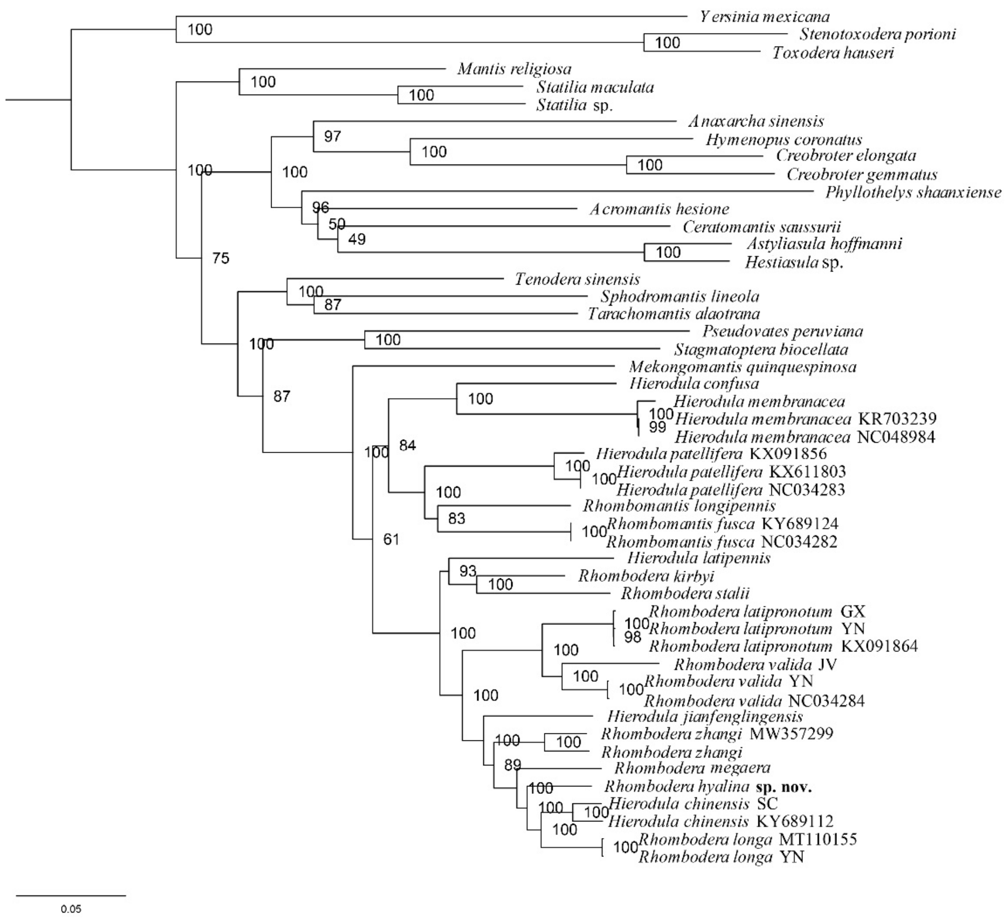

Figure S6. Topology of the ML analysis from PCGRNA-no partition dataset.

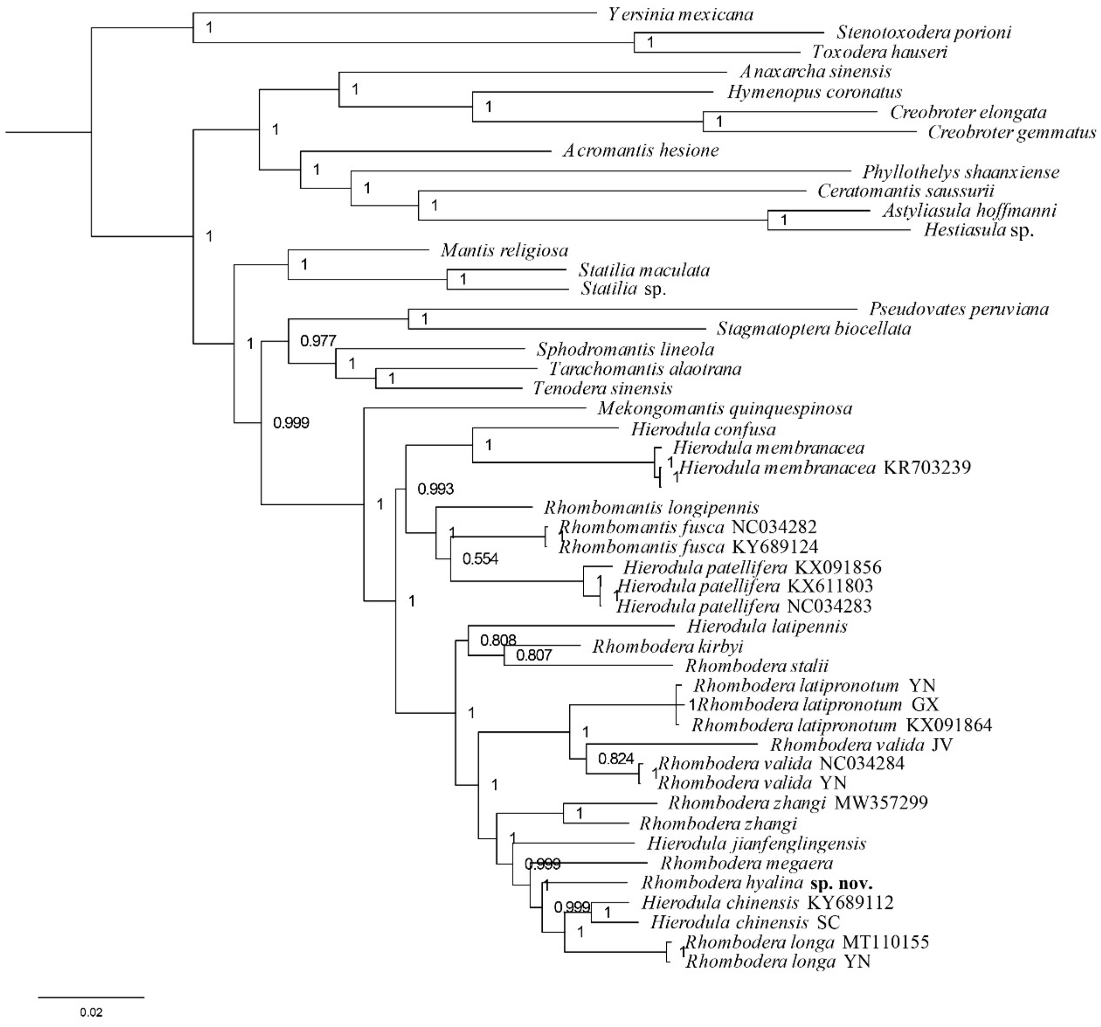

Figure S7. Topology of the BI analysis from AA-no partition dataset.

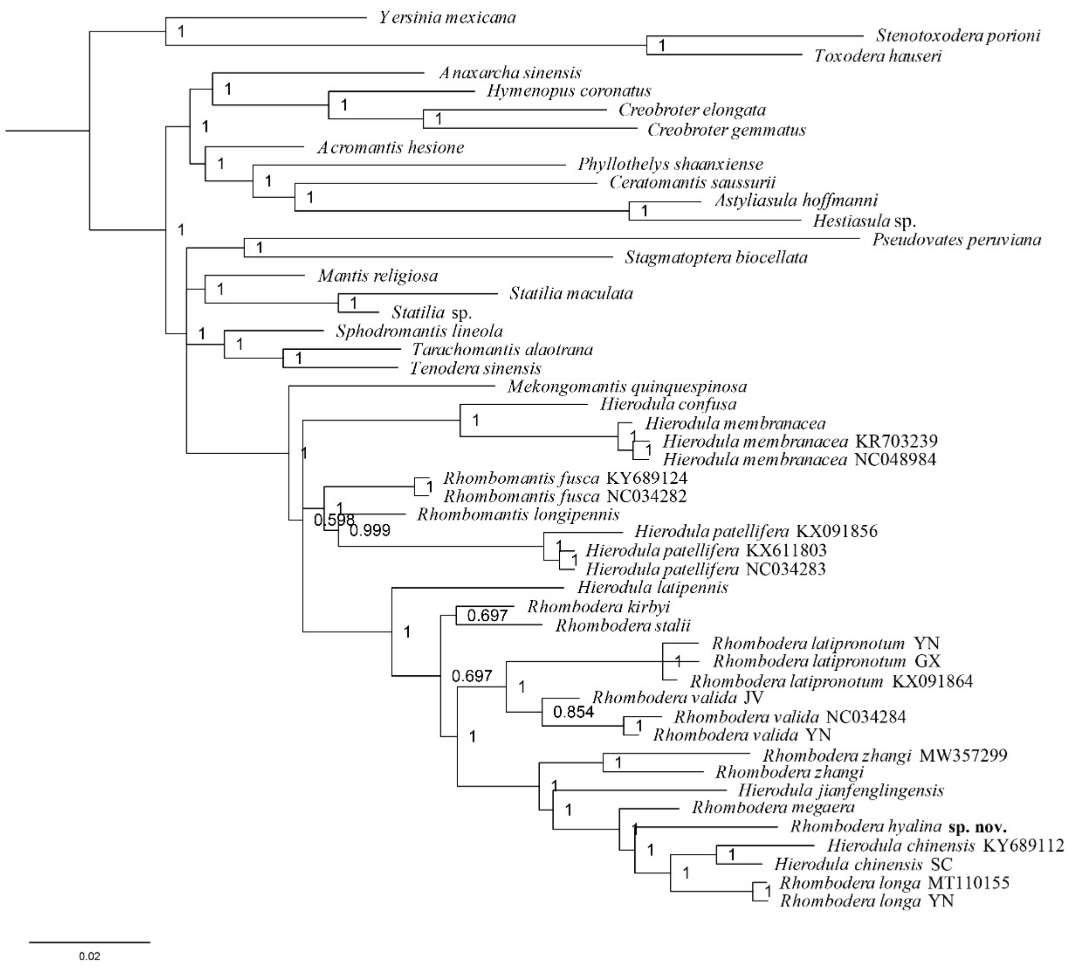

Figure S8. Topology of the BI analysis from AA-gene partition dataset.

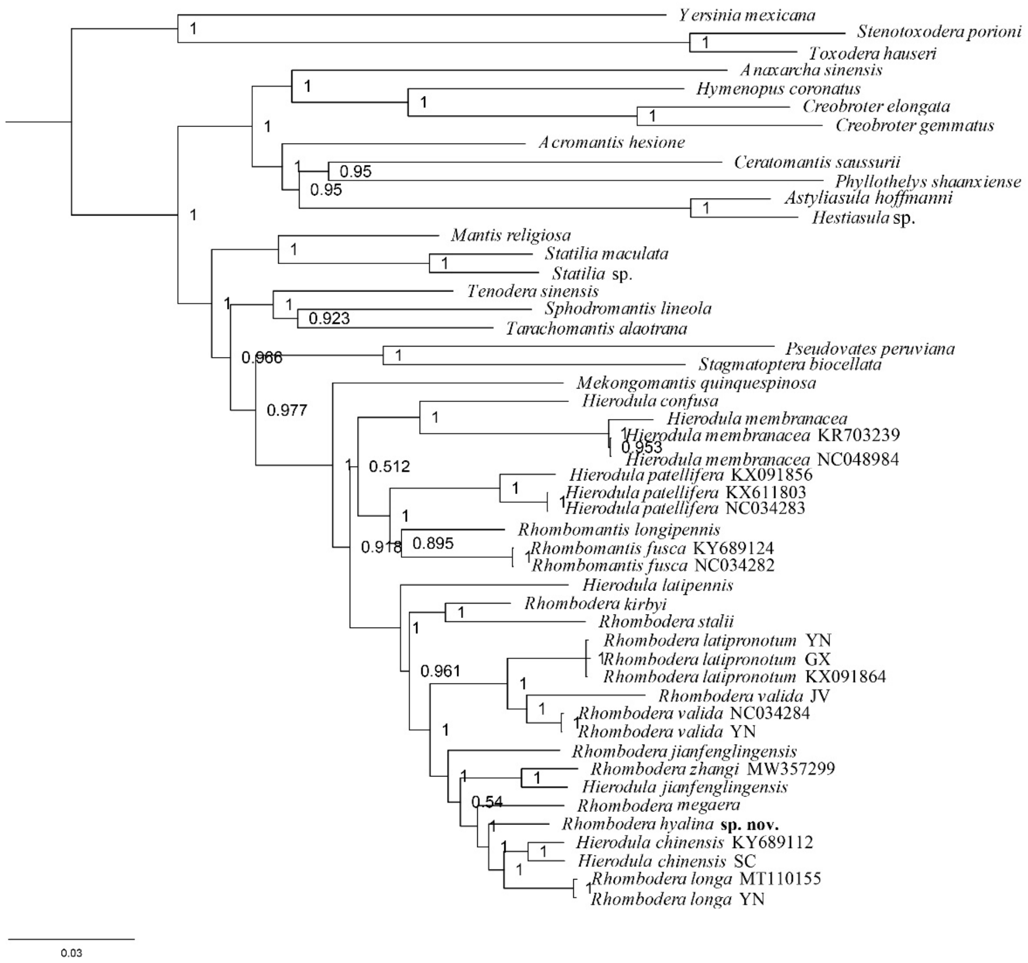

Figure S9. Topology of the BI analysis from PCG12RNA-gene partition dataset.

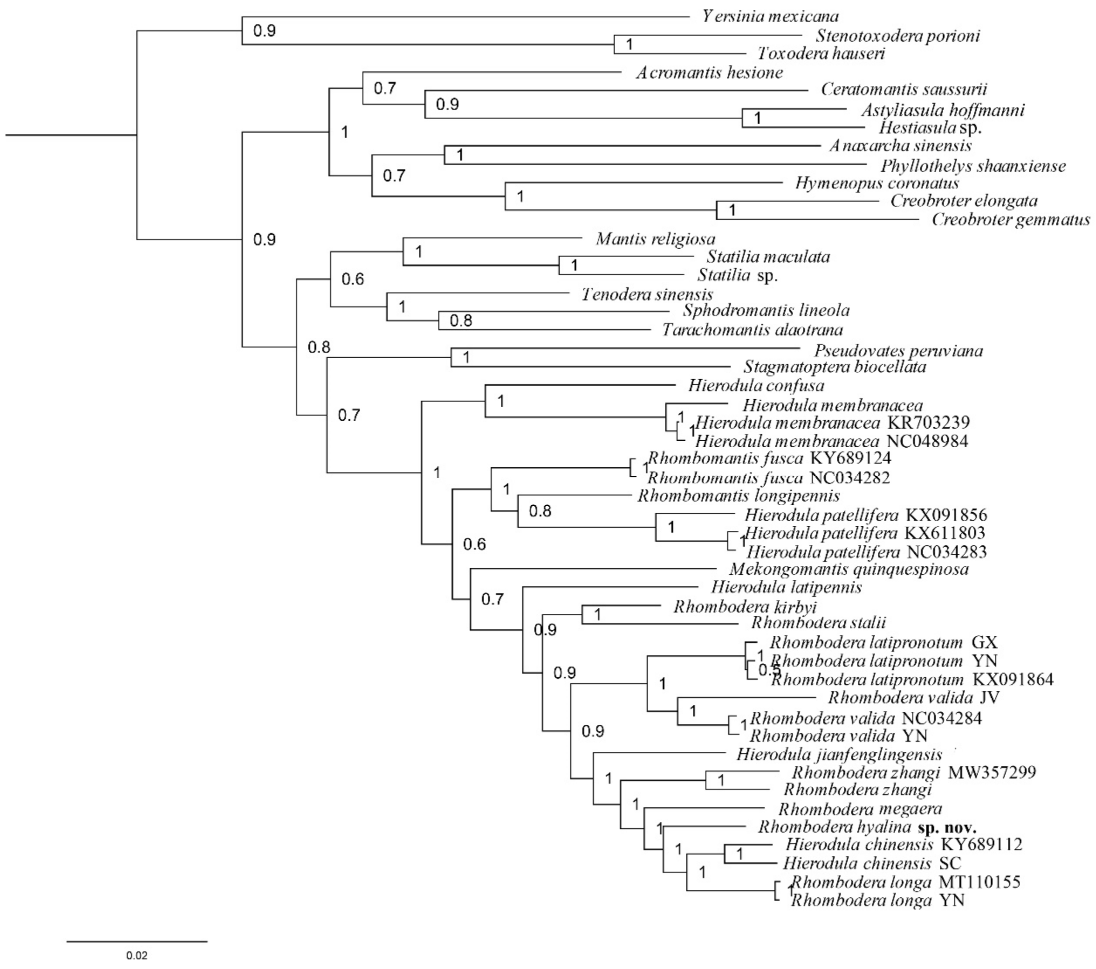

Figure S10. Topology of the BI analysis from PCG12RNA-no partition dataset.
